# Uroflowmetry nomogram in Iranian children aged 7 to 14 years

**DOI:** 10.1186/1471-2490-5-3

**Published:** 2005-03-16

**Authors:** Abdol-Mohammad Kajbafzadeh, Cyrus Ahmadi Yazdi, Omid Rouhi, Parvin Tajik, Parvin Mohseni

**Affiliations:** 1 Department of Pediatric Urology, Children's Hospital Medical Centre, Tehran University of Medical Sciences, Tehran, Iran; 2 Department of Biostatistics & Epidemiology, Faculty of Public Health, Tehran University of Medical Sciences, Tehran, Iran; 3 Department of Pediatric Nephrology, Children's Hospital Medical Centre, Tehran University of Medical Sciences, Tehran, Iran

## Abstract

**Background:**

As the voiding habits of Iranian children differs from other children because of some cultural and religious considerations, we aimed to establish normal reference values of urinary flow rates in Iranian children between 7 to 14 years of age.

**Methods:**

Eight hundred and two uroflowmetry studies were performed on children with no history of a renal, urological, psychological or neurological disorder, between the ages 7 and 14. Five hundred twenty five studies from 192 girls and 335 boys were considered in this study excluding the staccato/interrupted voiding pattern or voided volume less than 20 ml. The voiding volume, the maximum and average urinary flow rates were extensively analyzed.

**Results:**

The maximal and average urine flow rate nomograms were plotted for both girls and boys. Mean maximum urine flow rate was 19.9 (ml/sec) for boys and 23.5 (ml/sec) for girls with a mean voided volume of 142 (ml) for boys and 147 (ml) for girls. Flow rates showed a close association with voiding volume in both sexes. The maximum and average flow rates were higher in girls than in boys, and they showed a significant increase in flow rates with increasing age, where boys did not. The mean maximum urine flow rates (19.9 ml/sec for boys and 23.5 ml/sec for girls) were found to be higher in this study than other studies.

**Conclusion:**

Nomograms of maximal and average flow rates of girls and boys are presented in centile form, which can help the physician to evaluate the response to medical or surgical treatment and be useful for the screening of lower urinary tract disturbances in children, for a wide range of voided volumes.

## Background

Uroflowmetry is the most commonly used form of urodynamic testing. Simply stated, Uroflowmetry is the measurement of the rate of urine flow over time. It is easy to perform and it is a non-invasive test. Uroflowmetry alone is rarely able to determine the cause of a voiding dysfunction; however, it is extremely useful in selecting patients for more complex urodynamic testing [1]. It also provides an objective way of determining response to treatment of certain diseases [2]. Measured flow rates in children were first published by Kaufman [3] in 1957. He estimated normal flow rates at between 13 and 26 ml/sec with a lowest voiding volume of 100 ml.

Scott and Mcllhaney [4] measured maximum flow rate of 8–29 ml/sec at a voiding volume of over 100 ml. The lower limits of normal maximum and average urine flow rates are still unclear in both sexes. Recommended values for the lower limits of normal for the maximum urine flow rate in men range between 15 (ml/sec) [5] and 20 (ml/sec) [3]; in women, the values range between 12 [6] and 20 (ml/sec) [7].

Siroky et al. [8] constructed nomograms using data from a relatively small number of younger men. The nomogram of Jorgensen et al. [9] was restricted to older woman. All of the above mentioned nomograms were made for adults; one of the famous nomograms for children is Miskole's nomogram that was based on 433 micturitions. At maximum bladder fullness, the overall average flow rates in this study were 16 (ml/sec) in girls and 14 (ml/sec) in boys. The children were divided into three groups according to their body surface [10]. The aim of the present study was to establish the normal reference values in children of both sexes for maximum and average flow rates for a wide range of voiding volumes in Iran. Besides, there is no pediatric uroflowmetry nomogram for Iranian children and most uroflowmetry software use adult nomograms.

## Methods

This cross-sectional study was performed from April 2000 to February 2001 among 7–14 years old children. The study was approved by the Institutional Board Review and the Medical Ethics Committee of Tehran University of Medical Sciences. The samplings were done in 15 different schools throughout the city of Tehran.

We had many discussion sessions for children's parents as well as their teachers and other school staff, explaining the program and its harmlessness for the children. Children who wanted to take part in the program voluntarily and their parents filled in an informed consent were entered in this study.

Participants (n = 802) were told by their teacher to come to school with a full bladder in the morning. The mictiograph consisted of an electronic transducer installed in a specific toilet, with the recording equipment in a separate place. Testing was performed in a completely private toilet, lockable from inside. A nurse, skilled in clinical pediatric urology, helped us with the sampling, especially in girls' schools. Children started voiding when the operator told them. All children voided at maximum sensation of bladder fullness. No positional restriction during testing was applied and children were told to void in their habitual positions rather than specifying them to sit or stand. Uroflowmetry was performed using a uroflowmeter model Micromedics System/H, Model no. DPU- 441, Type 1001H 1995, England.

Uroflowmetry was performed on 802 children with no history of renal, urological, psychological or neurological disorder, 275 of the studies were excluded from the study because their voided volumes were less than 20 ml [11] or their flow curves were not bell shape (staccato or interrupted). For each subject data regarding maximum flow rate (Q-max), average flow rate (Q-ave), voided volume (vv), time to maximum flow, flow time and voiding time, as well as age, sex, height and weight was recorded.

Statistical analysis was performed using SPSS software for Windows version 11(SPSS Inc., Chicago, IL). A *P *value of 0.05 or less was considered as statistically significant. Several transformations of data were assessed and the goodness-of-fit tested to determine whether a linear, hyperbolic, parabolic or logarithmic function best described the relation between the maximum or average flow rates and the voided volume [12]. The quintile regression method was used to establish the percentile levels (5, 10, 25, 50, 75, 90, and 95). For presentation, the nomograms have been expressed in centile form and prepared for boys and girls separately. Differences were assessed for significance using Student's *t*-test.

## Results

We included Uroflowmetry studies of 527 children, 335 boys (63.6%) and 192 girls (36.4%) 7 to 14 years old. The mean age was 10.44 ± 2.25 years for boys and 9.73 ± 2.17 for girls.

The mean voided volumes were 142 ± 97.5 ml in boys and 147 ± 89.74 ml in girls. The mean maximum flow rate was 19.95 ± 10 (ml/sec) in boys and 23.52 ± 8.7 (ml/sec) in girls. The mean average urine flow rates were 8.1 ± 4.4 (ml/sec) for boys and 8.8 ± 4.2 (ml/sec) for girls. The minimum acceptable flow rates for boys and girls regarding age are shown in Table 1. Flow curves were bell shaped in 87% of all voids, staccato in 11% (32 boys and 26 girls) and intermittent in 2% (7 boys and 2 girls).

**Table 1:** Minimum acceptable flow rates for boys and girls between 7 to 14 years old (according to uroflowmetry parameters of healthy children who voided 100 ml or more).

Age	Boys		Girls	
	
	Q-max	Q-Ave	Q-max	Q-Ave
7–9	10	3.8	11	3.8
10–11	9.8	4	11.6	4.6
12–14	9.7	4.2	13.8	5.4

Maximum and average flow rates showed no significant increase with age in boys (Pearson's correlations: -0.11, *P *= 0.099 for Q-max and 0.09, *P *= 0.835 for Q-ave) but showed a statistically significant increase with age in girls (Pearson's correlations: 0.22 for Q-max, *P *= 0.001 and 0.27 for Q-ave, *P *= 0.002). So, in our study girls showed statistically significant increases in urine flow rates with age but boys did not. This increase was approximately 0.92 (ml/sec/year) for maximum urine flow rate and 0.51 (ml/sec/year) for average urine flow rate in girls between the ages 7 and 14.

Both sexes showed increases in maximum and average urine flow rates with increasing voided volume. The data sets for the study in both sexes allowed estimation of centiles for flow rates in the range of 20 to 350 ml. In boys, equations for the nomograms related flow rates with voided volume. These became:

(a) Square root (Q-max) = 2.05 + 0.2 × Square root (VV)

Root mean square error = 0.44 (H_0_: slope = 0, *P *< 0.001)

(b) Square root (Q-ave) = 1.36 + 0.12 × Square root (VV)

Root mean square error = 0.38 (H_0_: slope = 0, *P *< 0.001)

In girls, the association between voided volume and flow rate was best fitted by logarithmic function. The final equations for the nomogram graphs were then:

(a) Ln (Q-max) = 1.06 + 0.42 × Ln (VV)

Root mean square error = 0.36 (H_0_: slope = 0, P < 0.001)

(b) Square root (Q-ave) = - 0.67 + 0.74 × Ln(VV)

Root mean square error = 0.39 (H_0_: slope = 0, P < 0.001)

**Figure 1:**
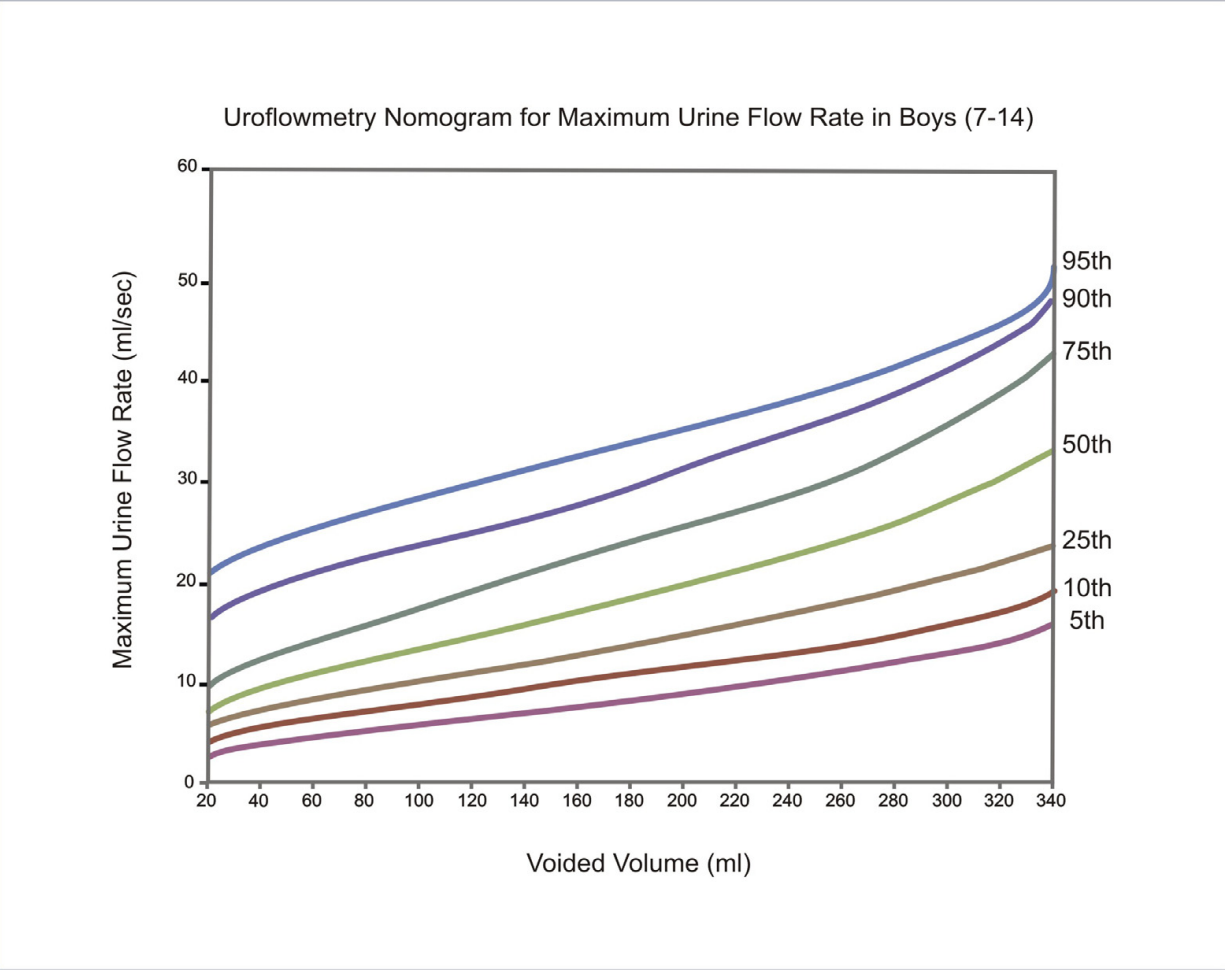
Uroflowmetry nomogram for maximum urine flow rates in Boys 7–14 in Iran

**Figure 2:**
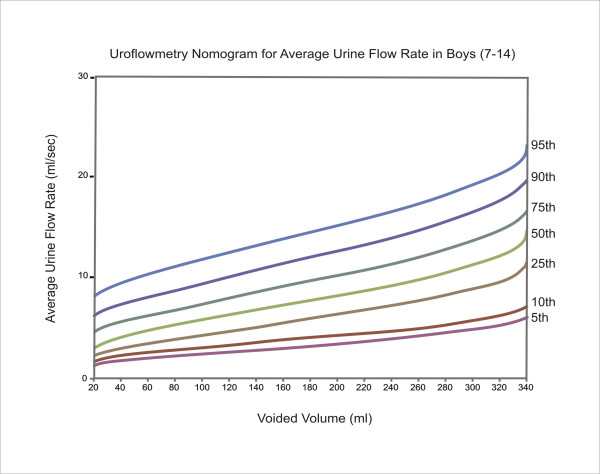
Uroflowmetry nomogram for average urine flow rates in Boys 7–14 in Iran

**Figure 3:**
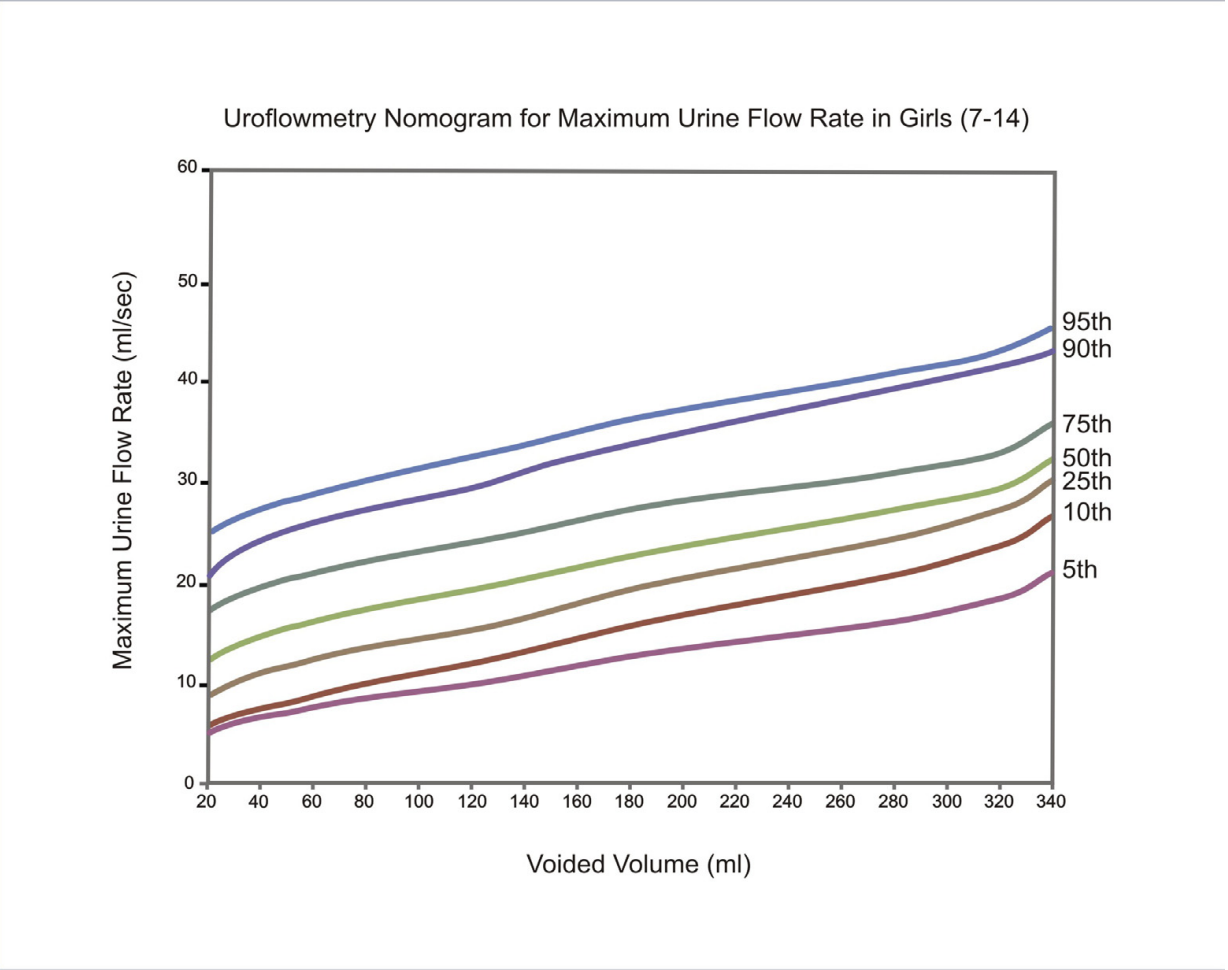
Uroflowmetry nomogram for maximum urine flow rates in Girls 7–14 in Iran

**Figure 4:**
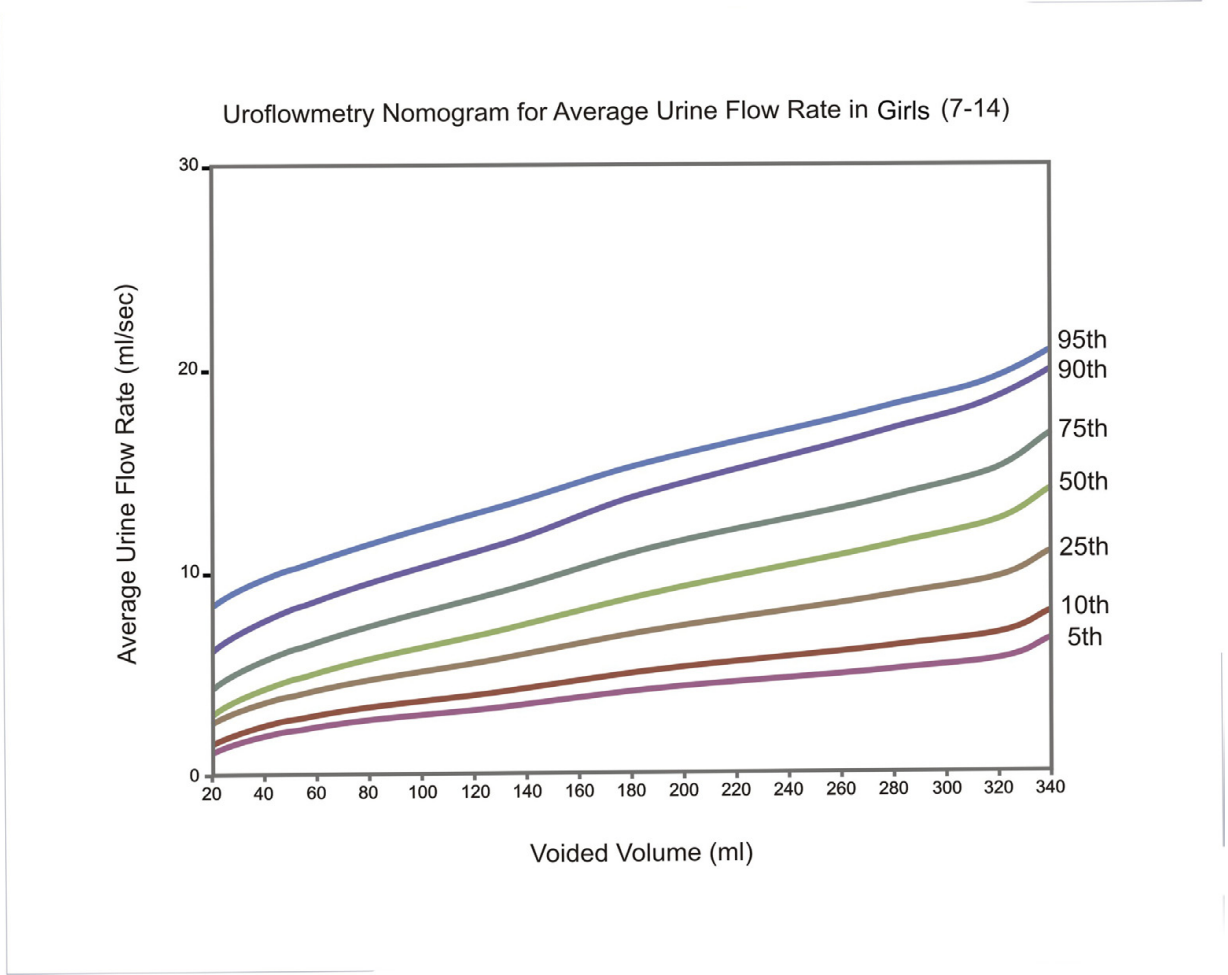
Uroflowmetry nomogram for average urine flow rates in Girls 7–14 in Iran

The equations for each centile are given in Table 2. Nomograms for the maximum and average urine flow rates for boys are shown in Figures 1 and 2, respectively. Figures 3 and 4 show the nomograms for the maximum and average urine flow rates for girls.

**Table 2:** The equations for nomograms The fitted parameters (5, 10, 25, 50, 75, 90, 95 percentiles) for the nomograms of maximum and average flow rates against voided volume for boys and girls. The parameter values substitute into the general equation: Sqrt (Q-max or Q-ave) = A × log (Sqrt (vv)) + B for Boys Log (Q-max) = A × log (vv) + B for Girls Sqrt (Q-ave) = A × log (vv) + B for Girls

Percentiles		Boys		Girls	
		
		Q-max	Q-Ave	Q-max	Q-Ave
5^th^	A	+0.155	+0.087	+0.55	+0.51
	B	+1.22	+0.82	-0.21	+0.48

10^th^	A	+0.164	+0.093	+0.63	+0.58
	B	+1.39	+0.92	-0.36	-0.65

25^th^	A	+0.189	+0.124	+0.54	+0.67
	B	+1.59	+0.99	+0.27	-0.69

50^th^	A	+0.229	+0.131	+0.38	+0.82
	B	+1.67	+1.25	+1.23	-1.09

75^th^	A	+0.245	+0.138	+0.28	+0.85
	B	2.11	+1.53	+1.90	-0.89

90^th^	A	+0.207	+0.14	+0.33	+0.86
	B	+3.06	+1.87	+1.87	-0.65

95^th^	A	+0.179	+0.134	+0.24	+0.73
	B	+3.77	+2.29	+2.39	+0.33

## Discussion

These nomograms were constructed to provide normal reference values in both sexes for urinary flow rate, covering a wide range of voided volumes and in centile form. Our nomogram is the only uroflowmetry nomogram for children in Iran. The use of nomograms avoids the dangers of referencing flow rates to any one voided volume. A maximum urine flow rate of 12 (ml/s) in men or 15 (ml/s) in women might fall just inside the fifth centile curve at 200 (ml) voided volume, although well outside the same curve at 400 (ml). The normality of these flow rates may then be interpreted quite differently at these 2 voiding volumes.

Siroky and colleagues were among the first to develop a nomogram that allowed uroflow to be corrected for voided volume. They also applied their nomogram to evaluation of men with bladder outlet obstruction. The authors stated that Q-max was a more sensitive indicator of outlet obstruction than Q-ave [8].

The Liverpool [13] nomogram was created from the micturitions of 331 normal adult men and 249 normal women. Men showed a significant decline in urinary flow rates with age, but women didn't show statistically significant variation in flow rates in this study. The authors believed that the voiding difficulties can be excluded in women by uroflowmetry, based on the 10th centile ranking that differentiates between women who are at low or high risk of having voiding dysfunction. Haylen and colleagues also believed that uroflowmetry could identify a subgroup of symptomatic men with high Q-max who are likely to have detrusor instability [13].

The use of statistical transformation by Gierup [14] and Jensen et al. [15] overcomes the problems created by inaccuracy when untransformed standard deviations were used. Churchill et al. [16] created nomograms for maximum urine flow rates for boys using regression analysis to fit functions to the data. Di Scipio [17] found only low correlations of age, height, weight and body surface area to average and maximum flow rates.

Abele and Krepler [18] reported that the flow rates of girls and boys were similar. Miskole [10] nomogram noted that the curves of 50% of the average and maximum flow rates were the same in both sexes when the voided volume was less than100 ml. But when the voided volume is more than 100 ml, the curves for the girls were higher. Gutierrez Segura's report [19] confirmed that the maximum and average flow increased with volume and age. Mean values were higher in girls than boys, and these differences were greater for maximum flow with larger volumes and older age except in the 12–14 year old group. He reported a normal flow pattern in 90% of healthy children, which is comparable to 87% Bell shape flow pattern in our study.

The mean maximum and average urine flow rates found higher in girls than in boys. This is interpretable by the girl's shorter urethra. The mean maximum urine flow rates (19.9 ml/sec for boys and 23.5 ml/sec for girls) were found to be lower in this study than those reported in western studies (Miskole [10]: Mean maximum urine flow rate 14 ml/sec for boys and 16 ml/sec for girls). This may be explained to some extent by the fact that because of religious considerations children have very early toilet training and parents force them to void every 1–2 hours in the toilet-training period to avoid wetting. These may cause a learned early sensation to void resulting in full sensation at lower bladder volumes. Low voided volume in healthy children is also confirmed in both sexes during 3 days voiding diary in this country (unpublished data).

## Conclusion

Nomograms in centile form are very useful for diagnosing urinary flow disturbances over a wide range of voided volumes. The present study provides reference values of maximal and average flow rates of normal boys and girls. Uroflowmetry is become a screening test for urinary problems in children.

## Competing interests

The author(s) declare that they have no competing interests.

## Authors' contributions

AMK conceived the study, participated in its design and coordination and finalized the manuscript. CAY and OR participated in design, collected the necessary data, reviewed the literature, and wrote a first draft of the manuscript. PT have reviewed the literature, edited the draft, revised the statistical analyses and nomograms and submitted the manuscript. PM participated in the design of the study. All authors read and approved the final manuscript.

## Pre-publication history

The pre-publication history for this paper can be accessed here:


